# Electrospun Polyvinyl Alcohol/d-Limonene Fibers Prepared by Ultrasonic Processing for Antibacterial Active Packaging Material

**DOI:** 10.3390/molecules24040767

**Published:** 2019-02-20

**Authors:** Weijie Lan, Xue Liang, Wenting Lan, Saeed Ahmed, Yaowen Liu, Wen Qin

**Affiliations:** 1 College of Food Science, Sichuan Agricultural University, Yaan 625014, China; weijie18728158690@163.com (W.L.); xue6liang17@163.com (X.L.); WTLan3253@163.com (W.L.); saeedahmed_mahar@yahoo.com (S.A.); 2 INRA, UMR408 Sécurité et Qualité des Produits d’Origine Végétale, F-84000 Avignon, France; 3 School of Materials Science and Engineering, Southwest Jiaotong University, Chengdu 610031, China; 4 California NanoSystems Institute, University of California, Los Angeles, CA 90095, USA

**Keywords:** polyvinyl alcohol, d-limonene, antibacterial active packaging

## Abstract

Novel fibers containing different ratios of PVA and d-limonene were fabricated using electrospinning for antibacterial active packaging applications. The PVA/d-limonene fibers were thoroughly characterized using a scanning electron microscope, fourier-transform infrared spectrometry, thermal gravimetry, differential scanning calorimetry, tensile tests, and oxygen permeability tests. The results of these analyses showed that the highest tensile strength and elongation at break values of 3.87 ± 0.25 MPa and 55.62 ± 2.93%, respectively, were achieved for a PVA/d-limonene ratio of 7:3 (*v*/*v*) and an ultrasonication time of 15 min during processing. This material also showed the lowest oxygen permeation and the best degradability and bacteriostatic properties of all samples.

## 1. Introduction 

Consumers are becoming increasingly health conscious and are eating more nutritious food. With this demands for high-quality and safe foods with natural flavor and long shelf life have greatly increased [1]. Thus, a variety of functional food packaging materials have been developed as an alternative to the traditional method of adding preservatives to the food to prolong its shelf life, examples include edible chitosan packaging materials [2], as well as the addition of clay nanoparticles to polymer blends to improve the mechanical and chemical properties of the packaging materials [3,4]. The incorporation of antioxidants or antibacterial agents in the packaging material reduces the oxygen content in the packaging environment, effectively slowing microbial growth [5]. Therefore, many food technology professionals advocate the inclusion of natural antioxidants in food packaging [6].

One such natural antibacterial agent is d-limonene, which is the predominant monoterpene in orange peel oil. It has significant chemopreventive and chemotherapeutic activity against chemically induced mammary, lung, and stomach cancer in rodents [7,8,9], while also inhibiting the growth of a wide range of bacteria and fungi [10]. Lisbalchin et al. reported the anti-bacterial function of limonene and showed that it was more biologically active than its isomers [11]. Limonene possesses excellent film-forming properties and can be applied as an edible surface coating to fruits and vegetables. Limonene coatings have been reported to limit fruit decay and delay the ripening of e.g., nectarines and strawberries [12,13]. The preharvest application of d-limonene solutions can effectively control postharvest fungal infection in green peppers [14].

Polyvinyl alcohol (PVA) is a biocompatible, biodegradable, and non-toxic water-soluble polymer prepared by the hydrolysis of polyvinyl acetate [15]. PVA has desirable physical properties, such as high elasticity, high degree of swelling in aqueous solutions, good emulsification and adhesion behavior, and excellent film-forming properties. Hence, electrospun PVA fibers have been receiving a lot of attention for use in many fields, including food packaging [16,17,18,19]. Ge et al. developed a novel food packaging material by immobilizing glucose oxidase in PVA/chitosan/tea extract membranes [20]; the immobilized glucose oxidase in electrospun membranes contributed to efficient deoxidization and inhibited microbial growth under low-oxygen conditions, thereby prolonging the shelf life of the packaged food. Wang et al. demonstrated an electrospinning technology for successfully combining antimicrobial peptide Ple with ultrafine PVA fiber mats [21]. They showed that Ple retained its antimicrobial activity when entrapped by the electrospun PVA fibers, indicating the potential for preventing foodborne pathogen contamination of foodstuffs and prolonging their shelf life. Although the application of PVA composite electrospun fibers has previously been demonstrated, to the best of our knowledge, there have been no reports of the fabrication of PVA/d-limonene composite fibers for food packaging applications.

Ultrasonic processing methods have been applied in many homogeneous and heterogeneous reactions for agitating or homogenizing solutions. Ultrasound can generate strong thermal [22], mechanical [23], and super-mixing effects [24], as well as cavitation phenomenon [25]. Cavitation is the formation, growth, and implosive collapse of bubbles in a liquid [26], which can further homogenize the particles on a microscopic scale [27]. Jackson et al. confirmed that ultrasound can affect the physicochemical properties of the polymers and increase the material solubility, resulting in compact net-like structures inside the films [28]. In addition, ultrasound can produce chemical changes, which have been exploited for many areas of chemical synthesis [29]. Li et al. used ultrasound irradiation to prepare PVAGOPs with six-member lactone rings and demonstrated that the interfacial interaction between GOPAs and PVA was effectively improved [30]. Liu et al. showed that increasing the ultrasound amplitude while processing sweet-potato-starch films resulted in an increase in light transmittance and tensile strength of the film [31].

The present study investigated the influence of d-limonene addition to electrospun PVA nanomaterials and evaluated ultrasonic processing of PVA/d-limonene electrospun fibers with different compositions (10:0, 9:1, 8:2, 7:3, 6:4, and 5:5 *v*/*v*). The electrospun PVA/d-limonene fibers were characterized using Fourier transform infrared spectroscopy (FTIR), scanning electron microscopy (SEM), thermal gravimetric analysis (TGA), and differential scanning calorimeter (DSC). Mechanical tests, soil burial tests, and oxygen vapor tests were performed to study the physical properties of fibers. The antimicrobial activity of the resulting fiber was evaluated by analyzing the inhibition of *Escherichia coli* and *Staphylococcus aureus* growth. 

## 2. Results and Discussion

### 2.1. Chemical Structure

**Figure 1:**
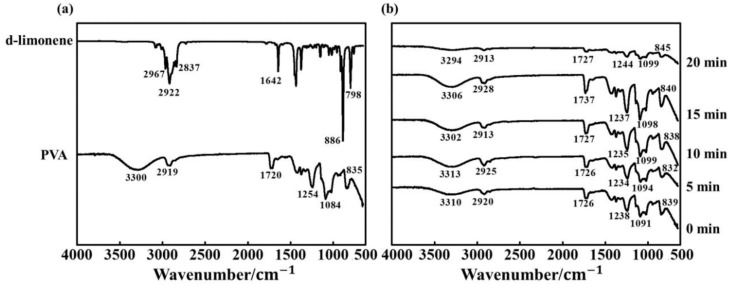
FTIR spectra of (**a**) pure PVA and pure d-limonene, and (**b**) PVA/d-limonene-5/5 fibers processed with different ultrasonication times.

ATR-FTIR was used to investigate the chemical interactions occurring in the PVA/d-limonene fibers, as shown in Figure 1a. The spectrum of pure d-limonene (Figure 1a) showed characteristic peaks at 798 and 886 cm^−1^ due to C-H bending, 1642 cm^−1^ due to C=C stretching, and 2837, 2922, and 2966 cm^−1^ due to C-H stretching [32]. The spectrum of the pure PVA fiber (Figure 1a) showed a band at 3300 cm^−1^, which was attributed to the O-H stretching vibration from the PVA hydroxyl groups in water; in addition, a band attributed to C-O stretching at 1084 cm^−1^ was observed. Bands at 2912 and 1720 cm^−1^ resulted from the stretching vibration and bending vibration of C-H, respectively, which have been previously reported for PVA [19]. 

The spectrum of the PVA/d-limonene composite fiber processed without ultrasound (Figure 1b); 0 min was very similar to that of the pure PVA sample, and the characteristic peaks of the d-limonene were not observed; this indicated that the incorporation of PVA with d-limonene did not significantly influence its structure and the chemical functionality of PVA was conserved. The addition of d-limonene weakened the intensities of the O-H absorption bands, which also shifted from 3300 cm^−1^ in the pure PVA fiber to 3310 cm^−1^ in the PVA/d-limonene composite fibers. This was attributed to the formation of new hydrogen bonds between the PVA and d-limonene molecules. As showed in Figure 1b, increasing the ultrasonic treatment time resulted in some changes in the FTIR spectra of the PVA/d-limonene composite fibers, especially after 20 min of treatment. The cavitation effect during ultrasonic processing is expected to have been greater with longer processing times, resulting in a greater number of hydrogen bonds between the PA and d-limonene being broken, while the super mixing effect intensified molecular motion, and further accelerated hydrogen bond breakage [33].

### 2.2. Thermal Properties

**Figure 2:**
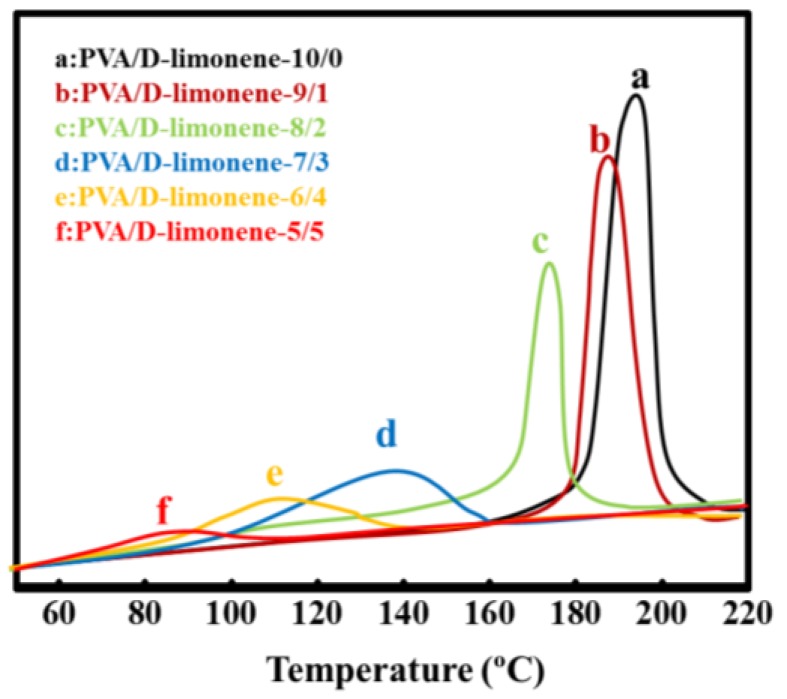
DSC curves of cooling.

PVA contains numerous hydroxyl groups, which from inter-, and intra-molecular hydrogen bonds, where the melting temperature (T_m_) is close to the decomposition temperature (~200 °C) [34]. Here, the thermal properties of PVA and PVA/d-limonene composite fibers were investigated using DSC and TGA. Table 2 and Figure 2 show the DSC curves obtained during cooling and DSC analysis results, respectively. The DSC curves indicate the good compatibility between PVA and d-limonene as the composite fibers only showed a crystallization temperature. The data in Table 1 shows that, with increasing d-limonene content, T_m_, T_C_ and the melting enthalpy (ΔH_m_) of the fibers decreased consistently, from 226.5 to 192.2 °C, 197.6 to 87.3 °C and from 44.38 to 25.31 J·g^−1^, respectively, this shows that modified by d-limonene is benefit for PVA thermoplastic processing. This was attributed to the d-limonene degrading hydrogen bonding both within PVA molecules and between PVA molecular chains. Hydrogen bonding between the PVA and d-limonene molecules also occurred, which impeded crystallization of the PVA molecules, resulting in lower T_m_ values. Forunati et al. also showed that the addition of limonene to PLA weakened the interaction between molecules, reduced the crystallinity, and decreased the thermal stability of PLA composite fibers [35]. 

**Table 1:** DSC analysis results of samples.

Samples	T_m_/°C	T_c_/°C	ΔH_m_/(J·g^−1^)
PVA/d-limonene-10/0	226.5	197.6	44.38
PVA/d-limonene-9/1	221.3	186.4	39.26
PVA/d-limonene-8/2	218.4	173.7	35.33
PVA/d-limonene-7/3	206.7	139.3	30.27
PVA/d-limonene-6/4	201.3	112.5	28.25
PVA/d-limonene-5/5	192.2	87.3	25.31

**Figure 3:**
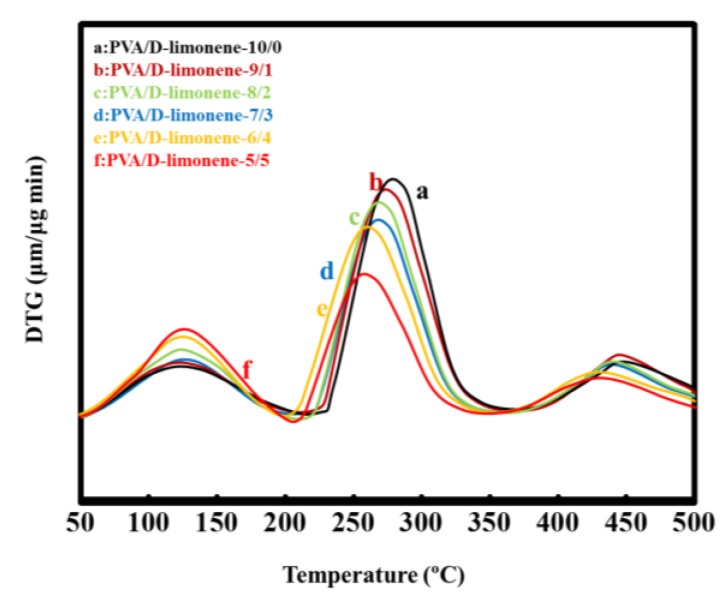
TGA curves of PVA/d-limonene fibers.

The TGA data for the PVA and PVA/d-limonene composite fibers are shown in Figure 3. The thermal decomposition of PVA occurs in three steps. The first stage occurs at 45–160 °C due to removal of moisture and physisorbed water molecules. The second stage at 180–320 °C is related to the thermal decomposition of the anhydrous compound; i.e., dissociation of the polymer chain and polymer melting. The third stage at around 300–400 °C is due to the cleavage of the PVA polymer backbone [36]. 

Figure 3 also shows that with increasing d-limonene content, the thermal stability of the polymer matrix decreased. Compared with pure PVA, the PVA/d-limonene composites showed stronger thermal degradation peaks (160~450 °C), indicating that the rate of thermal weight loss increased in this temperature range, while the d-limonene promoted thermal decomposition of PVA during the first stage. This could be due to the lower thermal decomposition temperature of PVA/d-limonene fibers than the pure PVA.

### 2.3. SEM Analysis 

**Figure 4:**
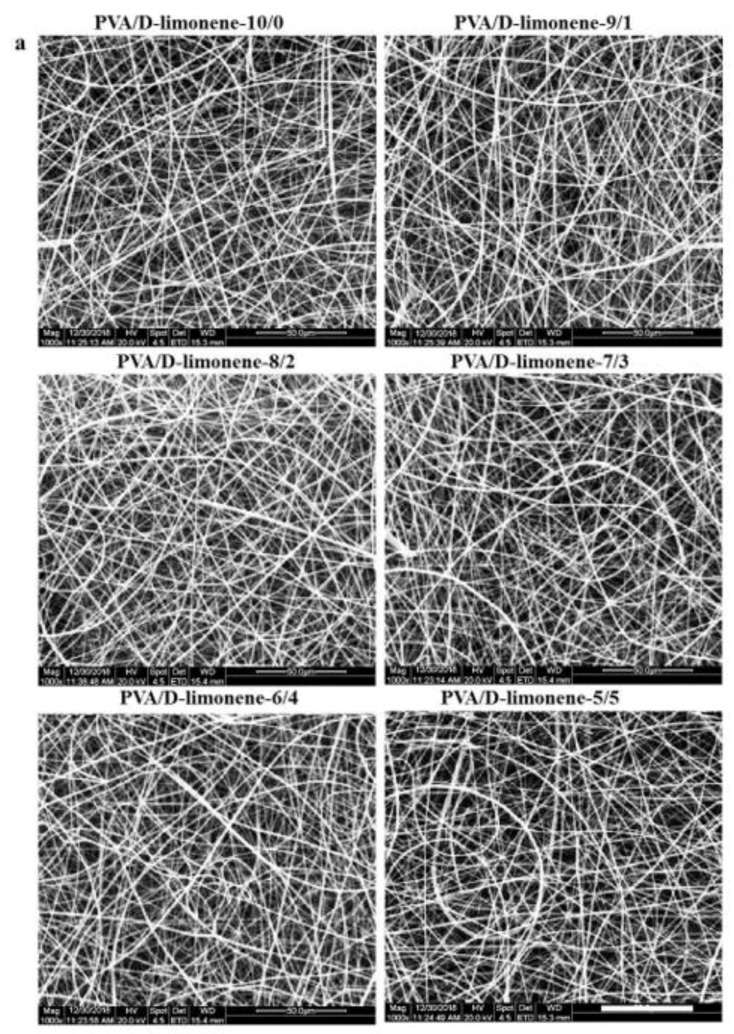

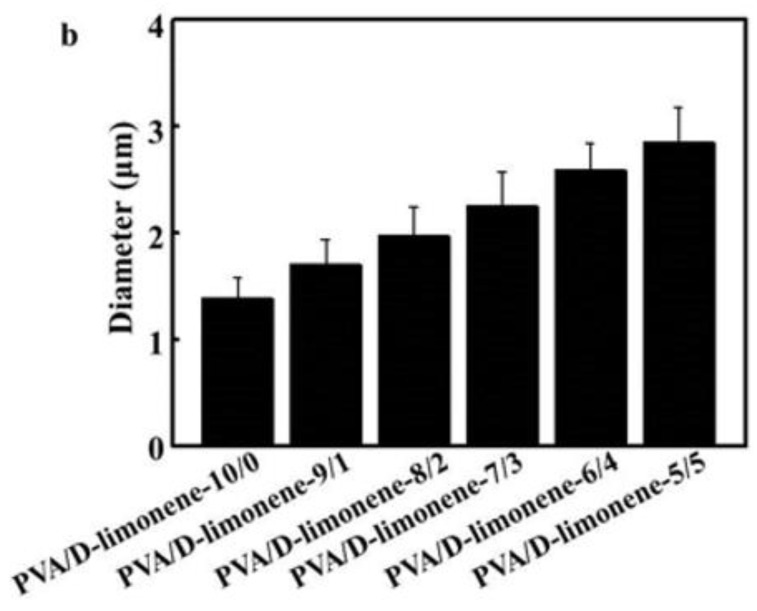
(**a**) SEM images of different PVA/d-limonene fibers; (**b**) average diameters of different fibers.

The morphologies of the PVA/d-limonene composite fibers are clearly visible in the SEM images (Figure 4a); the PVA fiber with no air bubbles, pores, cracks, or droplets are observed. When the d-limonene content was increased, the morphology of the composite electrospun fibers did not change significantly, and good structural integrity, smoothness, and flatness were observed. This might be due to the high compatibility of the two polymers, as previously described [15]. As shown in Figure 4b, the PVA fibers had a small average diameter (1.45 ± 0.35 μm). Accordingly, increasing the concentration of d-limonene in composite fibers sharply increased the fiber diameter; PVA/d-limonene composite fibers had a greater average diameter (range 1.75 ± 0.43 μm~2.84 ± 0.61 μm), probably because of a higher evaporation point and dielectric constant (176 °C; 2.3, respectively) of d-limonene, thus slower evaporation takes place, resulting in fibers with higher average diameter, as reported in previous studies [37]. The increase in fiber diameter suggests that the bioactives were efficiently encapsulated and distributed within the microfibers.

### 2.4. Mechanical Properties

**Figure 5:**
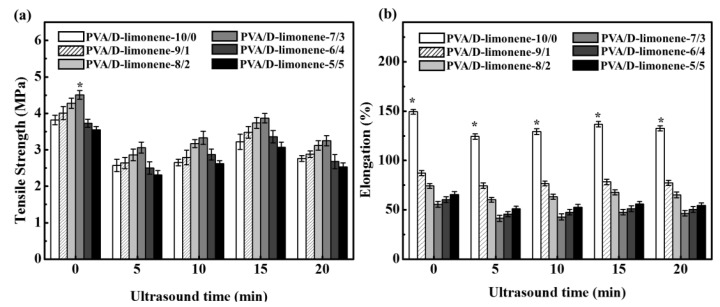
(**a**) Tensile strength and (**b**) elongation of PVA/d-limonene fibers with different volume ratios and ultrasonic processing times.

For the same ultrasonic processing time, the addition of d-limonene clearly changed the tensile strength and elongation at break of the PVA/d-limonene fibers, as shown in Figure 5a and Figure 5b, respectively. The pure PVA fibers had a low tensile strength of 3.82 ± 0.13 MPa and high elongation at break of 149.32 ± 2.23%. With increasing d-limonene content, the tensile strength increased gradually, reached a maximum value for a PVA/d-limonene content of 7:3 (*v*/*v*), then decreased. With increasing d-limonene content, the elongation at break decreased rapidly, reached a minimum value for the PVA/d-limonene = 7:3, then increased slowly. Considering the physical structure, the addition of d-limonene resulted in narrower fibers and a denser nanostructure, which increased the friction between the fibers, resulting in a decrease in slip. Therefore, the tensile strength initially increased and the elongation at break decreased with the addition of d-limonene. With further addition of d-limonene, the relative fiber content decreased, resulting in adhesion and filamentation, which was observed as a decrease in the tensile strength of the PVA/d-limonene fibers to values even lower than that of the pure PVA. Considering the molecular interactions, the tensile strength is related to the binding force between molecular chains, while the elongation at break is related to molecular chain movement [38]. PVA is a semi-crystalline polymer in which both crystalline and amorphous regions coexist [39]. With the addition of d-limonene, some short chains of d-limonene are intercalated into the PVA macromolecular chains, which impede stretching of the PVA molecular chains, resulting in a decrease in the elongation at break. Moreover, due to the plasticization effect of the d-limonene, a significant increase in the mobility of the chains and subsequent increase in the flexibility of PVA fibers occurs, which increases the elongation at break of the composite fibers.

For the same d-limonene content, the ultrasonic processing resulted in an overall decrease in the tensile strength and elongation at break of the PVA/d-limonene fibers. With increasing ultrasonic processing time, the tensile strength and elongation at break increased gradually, reached the highest values after 15 min, then decreased slightly. 

### 2.5. Oxygen Permeability

**Figure 6:**
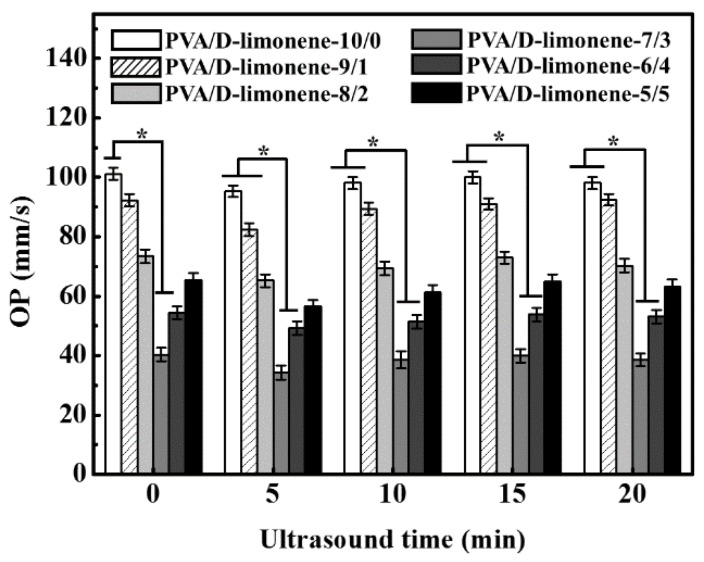
Oxygen permeability of different fibers with different ultrasonic times.

The oxygen permeability (OP) behavior is very important for packaging materials used for food preservation applications [40]. Figure 6 shows the measured OP values of the PVA/d-limonene composite samples. For the same ultrasonic processing time, the OP values first decreased and then increased with increasing d-limonene content. This was attributed to the smaller fiber diameter after d-limonene addition; however, when the d-limonene content increased, the fiber diameter increased and the uniformity of the samples was enhanced. In theory, larger fiber diameters result in larger OP values for fibers. Therefore, the PVA/d-limonene-7/3 sample showed the lowest OP value of 40.27 ± 2.32 mm/s of all samples. Moreover, with increasing ultrasonic processing time, the OP first decreased slightly, and then increased after 15 min of ultrasound time. During a certain processing time, the ultrasonic waves cause the molecules to rearrange and form a dense, uniform network structure, which reduces the oxygen transmission rate of the composite fibers to some extent. However, longer ultrasonic treatment induced more intense molecular motion, resulting in a disordered structure that impedes adhesion between fibers, creating more defects that allow oxygen to pass through the fiber.

### 2.6. Biodegradation of the Composite Fibers

**Figure 7:**
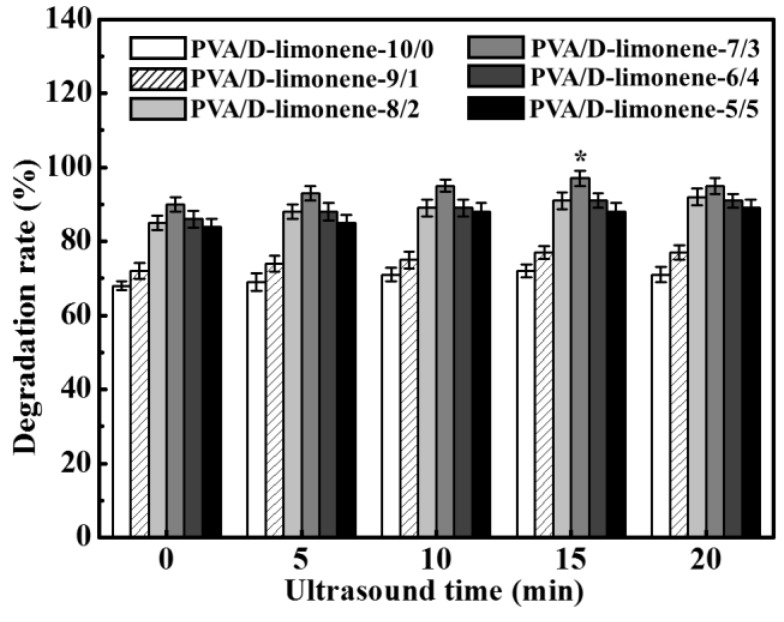
Degradation percentage of different fibers with different ultrasonic times.

PVA fiber has good water absorption due to the large amount of hydrophilic groups, where the degradation products are water and carbon dioxide. The biodegradation rate of PVA/d-limonene composite fibers was determined from their weight loss after 2 d of burial in soil. The degradation rates as a function of d-limonene content and ultrasound time are shown in Figure 7. The degradation rate increased with increasing d-limonene content for the same ultrasonic processing time. The pure PVA showed the lowest degradation rate (68.57 ± 2.47%) of all samples after 2 d storage. Azaliari et al. [41] also reported that pure PVA showed the lowest degradation rate after being buried in soil. The sample with the highest d-limonene content (PVA/d-limonene-7/3) had the highest degradation rate of 90.03±1.98%. PVA is soluble in water and d-limonene is inherently biodegradable. The degradation of PVA/d-limonene was due to the metabolic activity of microorganisms in the soil that consume the constituents of the degraded material as a nutrient source and the good water solubility of PVA. d-limonene is not easily soluble in water. With the increase of d-limonene content, d-limonene is gradually wrapped on the outer surface of the PVA/d-limonene fibers, which causes the water solubility of the composite fiber to decrease, resulting in a decreased in degradation rate. Figure 7 also shows that with increasing ultrasonic processing time, the degradation rate increased until 15 min, then stabilized. This was attributed to cavitation effects during ultrasonic processing which enhanced the uniformity of the fibers and hence, their degradability. 

### 2.7. Antibacterial Properties

**Figure 8:**
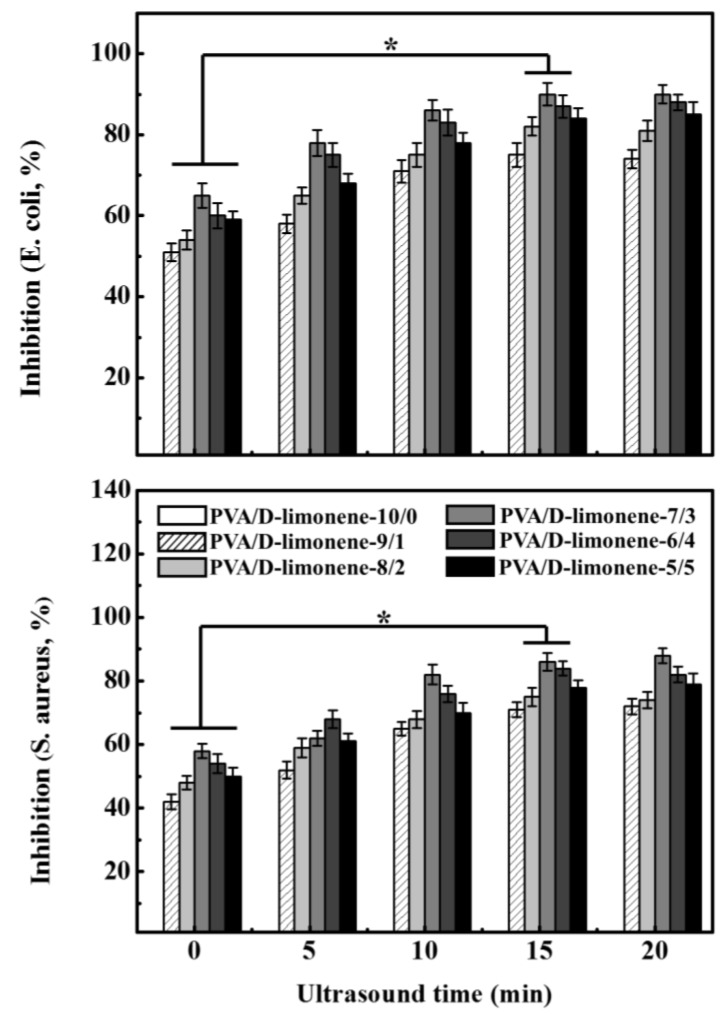
Effect of PVA/d-limonene fibers on the growth inhibition (%) of *S. aureus* and *E. coli*.

Figure 8 shows the antibacterial properties of the PVA/d-limonene fibers with different d-limonene contents against *E. coli* and *S. aureus*. The growth inhibition of both types of bacteria increased with the addition of d-limonene, where the antibacterial effect was better for *E. coli* than *S. aureus*. Rahyour et al. [42] previously showed that the principal antibacterial mechanism of d-limonene is against the cytoplasmic membranes of the microorganisms, which results in the loss of membrane integrity, dissipation of proton-motive forces, and inhibition of the respiratory enzymes. Therefore, with increasing d-limonene content, the composite fibers showed gradually increasing bacteriostatic effects, where PVA/d-limonene-7/3 showed the highest antibacterial value for *E. coli* and *S. aureus* of 65 ± 2.11% and 58 ± 3.28%, respectively, without ultrasonic processing. However, with the further increase of d-limonene, the antibacterial result of PVA/d-limonene fibers showed a decreasing trend. This is related to the morphology of the fibers. As the d-limonene content increases, the fibers became cohesive, the fiber distribution was uneven and accompanied by the appearance of small beads, which leads to the uneven distribution of d-limonene, then apparently causes the antibacterial effect appear to decrease. Ultrasonic processing improved the antibacterial properties of the fibers to some extent. The bacteriostatic activity first increased and then stabilized after 15 min. This is attributed to the d-limonene being dispersed homogeneously in the PVA solution via ultrasonic processing, which increased the d-limonene content per unit area of the fiber, increasing its contact with the bacteria and enhancing the antibacterial properties. This is consistent with our previous study that showed that ultrasonic processing can improve the antibacterial properties of fibers [43].

## 3. Materials and Methods

### 3.1. Materials

The PVA used in this study (Mw = 7.6 kDa, Mw/Mn = 1.32) was supplied by Shenzhen Esun Industrial Co., Ltd. (Shenzhen, China), and we used d-limonene (97% pure; Mw = 136.23) from Chengdu Kelong Reagent Co. (Chengdu, China). All other chemicals and solvents were of reagent grade or higher purity, and were purchased from Yan’an Wanke Reagent Co. (Ya’an, China), unless otherwise indicated.

### 3.2. Electrospinning PVA/d-limonene Composite Fibers

To prepare the electrospinning solutions, 10 g of PVA powder was added to 90 g of deionized water at room temperature [44]. The powder was allowed to swell for 1 h, after which the resulting mixture was gradually warmed to 90 °C. The d-limonene solution (1:10 wt % d-limonene: ethyl alcohol) was added to the aqueous PVA mixture. Table 2 shows the compositions and processing conditions for the different fibers prepared in this study. A range of PVA/d-limonene mass ratios was prepared (10:0, 9:1, 8:2, 7:3, 6:4, and 5:5 *v*/*v*). Then, the mixtures were stirred constantly at 800 rpm for 15 min. The solutions were treated with ultrasound in a TOPSNIC ultrasound liquid processor (UP-400 series, Tehran, Iran) for 0, 5, 10, 15, or 20 min, with an ultrasonic frequency of 40 Hz and power of 50 W in order to investigate the effect of ultrasound on the formation of the fibers. During the experiment, the ultrasonic probe horn was inserted directly into the liquid media. 

For the electrospinning experiments, the solutions were added to a circular metal capillary with an inner diameter of 0.7 mm using a 5 mL syringe. The flow rate was kept at 0.2 mL/h using a syringe pump (Zhejiang University Medical Instrument Company, Hangzhou, China). The distance between the capillary tip and collector was set at 20 mm and a voltage of 18 kV was applied using a high-voltage station (Tianjin High Voltage Power Supply Company, Tianjin, China). A grounded rotating mandrel covered by aluminum foil used to collect the fibers during rotation. 

**Table 2:** Summary of the samples with different PVA/TP ratios and ultrasonication times.

Sample Number	Sample Name	PVA/d-limonene Ratio	Ultrasonication Time
1	PVA/d-limonene-10/0-0	10/0	0
2	PVA/d-limonene-10/0-5	10/0	5
3	PVA/d-limonene-10/0-15	10/0	15
4	PVA/d-limonene-10/0-20	10/0	20
5	PVA/d-limonene-9/1-0	9/1	0
6	PVA/d-limonene-9/1-5	9/1	5
7	PVA/d-limonene-9/1-15	9/1	15
8	PVA/d-limonene-9/1-20	9/1	20
9	PVA/d-limonene-8/2-0	8/2	0
10	PVA/d-limonene-8/2-5	8/2	5
11	PVA/d-limonene-8/2-15	8/2	15
12	PVA/d-limonene-8/2-20	8/2	20
13	PVA/d-limonene-7/3-0	7/3	0
14	PVA/d-limonene-7/3-5	7/3	5
15	PVA/d-limonene-7/3-15	7/3	15
16	PVA/d-limonene-7/3-20	7/3	20
17	PVA/d-limonene-6/4-0	6/4	0
18	PVA/d-limonene-6/4-5	6/4	5
19	PVA/d-limonene-6/4-15	6/4	15
20	PVA/d-limonene-6/4-20	6/4	20
21	PVA/d-limonene-5/5-0	5/5	0
22	PVA/d-limonene-5/5-20	5/5	5
23	PVA/d-limonene-5/5-15	5/5	15
24	PVA/d-limonene-5/5-20	5/5	20

### 3.3. Characterization of Fibers

The morphologies of PVA/d-limonene composite fibers were investigated by a scanning electron microscope (SEM, FEI Quanta 200, Philips, The Netherlands). The electron accelerating voltage for SEM was 20.0 kV with and a Robinson detector after 2 min of gold coating in order to minimize the charging effect. The diameter of fibers was determined manually from SEM images using ImageJ as described previously [15]. 

TGA was performed at a heating rate of 10 °C/min under a nitrogen atmosphere (TGA-Q500, TA Instruments, New Castle, DE, USA). DSC experiments were performed using a DSC Q100 module (DSC Q100 module, TA Instruments, New Castle, DE, USA) under a nitrogen flow over a temperature range of 0–250 °C at a heating rate of 10 °C/min. After a cooling step, a second heating step was performed. Attenuated total reflectance FTIR (ATR-FTIR) spectrometry (Bruker Gmbh, Ettlingen, Germany) was used to identify the chemical structures of the PVA/d-limonene composite fibers and investigate possible interactions between their components. The samples were scanned at a resolution of 4 cm^−1^ over the range of 500–4000 cm^−1^, with an aperture setting of 6 mm, scan frequency of 2.2 kHz, background scan time of 32 s, sample scan time of 32 s.

The uniaxial tensile properties of fibrous mats were measured using a mechanical testing machine (Instron 5567, Boston, NY, USA) at a cross-head speed of 2 mm/min with a 5-N load cell. as described previously [15]. The fibrous mats were punched into small strips (40 × 20 × 1 mm^3^) and the uniaxial tensile properties were determined from five separate measurements of each sample, while the elongation was obtained from stress-strain curves. Permeability tests were performed using a PERME TM OX2/231 permeability tester from Labthink Instruments Co., Ltd. (Jinan, China). Nitrogen was used as the oxygen carrier gas (relative humidity ~50%) at a temperature of 23 °C; the oxygen and nitrogen flowrates were 20 and 10 mL/min, respectively.

### 3.4. Biodegradation of Fibers

Standard SR EN ISO 846/2000 was adapted in order to evaluate the biodegradability of the composite fibers in natural active soil. Rectangular samples with a length of 10 cm and width of 8 cm were used. The samples were weighed and then buried in the soil under aseptic conditions. In order to determine the variation in weight, the sample batches were stored in a desiccator at ambient temperature until the weight of each sample reached a constant value. The samples were weighed both at the beginning (m_1_) and end of the test period (m_2_) and the percentage degradation was calculated using [(m_1_ − m_2_)/m_1_] × 100 (%). The samples were buried in soil for 2 d to a depth of about 10 cm to allow them to degrade naturally. Sample incubation was performed at a temperature of 45 °C for 2 d. Three samples for each PVA/d-limonene mass ratio were measured, and the data shown here are average values.

### 3.5. Antimicrobial Tests 

The antimicrobial properties of the fibers were evaluated using a shake-flask culture method [45] with *Escherichia coli* (*E. coli*, ATCC 25922) and *Staphylococcus aureus* (*S. aureus*, ATCC 29523). Firstly, *E. coli* and *S. aureus* were cultured in BHI and LB media and shaken at 37 °C for 24 h. Then, the bacterial suspension was diluted in PBS to 1–5×10^7^ CFU/mL. Fibers (100 ± 2 mg) were immersed in 20 mL of PBS containing 200 μL of 1–5 × 10^7^ FU/mL of bacterial suspension were placed in a 100 mL flask, while PBS (200 mL) containing 200 μL of 1–5 × 10^7^ CFU/mL of bacterial suspension was used as a control. All flasks were incubated in an incubator-shaker at 37 °C for 48 h. Next, 100 μL of the bacteria suspension was spread on a nutrient agar plate, which was incubated at 37 °C for days. Then, the number of colonies was counted and the bacteriostatic rate was calculated using [(W_1_ − W_2_)/W_1_] × 100 (%), where, W_1_ is the number of colonies formed on the PVA/d-limonene sample and W_2_ is the number of colonies formed on the fibers.

### 3.6. Statistical Analysis

Multiple samples were tested and the results are reported as the mean ± standard deviation. The data were analyzed using a one-way analysis of variance (ANOVA) with SPSS software (IBM, Armonk, NY, USA). The value of *p* < 0.05 was considered significant.

## 4. Conclusions

In this study, PVA/d-limonene composite fibers were successfully prepared via electrospinning. The effects of ultrasonic processing time on the properties of the composite fibers were studied. Over a certain range, ultrasonic processing resulted in more homogeneous fibers and affected their properties. fiber with a PVA/d-limonene volume ratio of 7/3 and an ultrasonic processing time of 15 min. The results of these analyses showed that the highest tensile strength and elongation at break values of 3.87 ± 0.25 MPa and 55.62 ± 2.93%, respectively, were achieved for a PVA/d-limonene ratio of 7:3 (*v*/*v*) and an ultrasonication time of 15 min during processing. In addition, we identified optimal processing conditions to produce films with good mechanical properties, minimized oxygen permeability, and good antibacterial activity.
